# On the Dislocation of the Lower Jaw

**Published:** 1850-08

**Authors:** 


### SELECTIONS

FROM

### AMERICAN AND FOREIGN JOURNALS.

---

*On the Dislocation of the Lower Jaw:* by M. Nelaton.—The generally received theory of luxation of the jaw, founded on the writings of our highest authorities, is, that, once produced, the condyle of this bone becomes placed in front of the transverse root of the zygomatic apophysis; and is maintained there, according to some, by muscular contraction; according to others, by the resistance which the projection of the apophysis offers. M. Malgaigne was one of the first to contest the validity of this hypothesis, and showed the ease with which the condyle could be carried before the tubercle, and returned to its cavity; and that, in fact, this is a normal movement, which any one may effect on himself. M. Nelaton has undertaken some researches upon the subject; and the following is the anatomical explanation of the dislocation which he offers:

"In front of the temporo-maxillary articulation is the temporo-zygomatic excavation, in which the coronoid process is lodged when the mouth is closed. Before and behind this excavation two eminences are found, the posterior being formed by the transverse apopysis of the zygomatic arch, and the anterior by the junction of the malar with the superior maxillary bone. At the lower part of the suture uniting these two bones, a tolerably prominent tubercle is formed, bounded within by the furrow formed by the smooth border of the malar process of the maxilla, and frequently on the outer side by a little, elongated, nearly oval *fossette*, to which we shall have again to refer. This tubercle, which may be called the *malar tubercle*, is about a centimetre distant from the coronoid process; in its place we have sometimes seen a plane surface, or even a more or less deep furrow; but as a general rule it exists.

"The coronoid process also requires our careful attention. In fact, as M. Chassaignac has remarked, in his memoir on the disarticulation of the jaw, this process presents some well-marked individual differences. Thus, in some subjects, it is so short as to scarcely rise to a level with the condyle, but it is in others much elongated. In some it is directed almost directly upwards, and in others obliquely outwards, nearly to the zygomatic arch; sometimes it stretches forwards, far away from the condyle, and at others inclines backwards towards it.

"In my experiments upon the dead body, I found—*first*, as already shown by M. Malgaigne, that if the condyle is carried forward, without going beyond the point which the laxity of its capsule allows it to attain, the displacement at once disappears as soon as the jaws are closed, the tubercle of the transverse apophysis offering no obstacle to the retrocession of the condyle; and, *secondly*, if the anterior part of the capsule is cut or torn, so as to allow the condyle to quit it and advance some millimetres, the displacement becomes permanent, not, as is generally believed, on account of the tubercle, or the contraction or tension of the muscles, but because *the extremity of the coronoid process is projected against the lower and anterior angle of the malar bone, and is lodged in the little fossette we have described as often there existing.*

"The contact of the apex of the coronoid process with the malar bone, seems to me, then, to be an indispensable condition for the production of a true dislocation; and to effect this, the displacement need not be very great, for it suffices if the condyle advances three or four millimetres. The external lateral ligament remains uninjured, the capsule being alone torn at its anterior part. The interarticular cartilage accompanies the condyle in its displacement, or remains beneath the transverse apophysis, according to whether the rupture takes place above or below its anterior edge. When the luxation is produced by external violence, the coronoid process probably ruptures some of the fibres of the masseter or temporal, and, becoming lodged in the substance of these muscles, the difficulty of reduction is increased." (pp. 399–401.)

This relative position of the parts has not passed entirely unobserved; for Monro the elder believed that, when the reduction was not affected, the jaw was gradually raised, until the coronoid process came in contact with the malar bone; and S. Cooper incidentally mentions the same disposition of parts; but neither of them employed the fact in the explanation of the mechanism, the pathological anatomy, or the treatment of the accident. From what has been said, it will be perceived that this dislocation must be a rare one, depending so much as it does upon the size and direction of the coronoid process. When this extends far backwards, the jaw must be thrown forcibly forwards to allow of the contact being produced, and in some subjects its production would be probably impossible. On the contrary, the projection of the process forwards would render the production of the accident easier; and if this disposition were in excess, the luxation might be produced, without any violence, by the sole action of the depressors of the jaw. Such dislocations would be very apt to recur, as, in fact, the history of the accident shows to be the case. The present theory explains the symptoms of the displacement, and the difficulties of its reduction.

Since the paper has been read, several members have communicated cases to the Society, confirmatory of the views set forth in it, and showing the assistance in the reduction of the dislocation obtainable by making pressure below the malar bone. In the 37th volume of the "Bulletin de Thérapeutique" (p. 404,) a case is narrated, that recently came under M. Nelaton's care. It occurred to a lady who dislocated her jaw while yawning, and all efforts at reducing it in the ordinary manner had failed, when M. Nelaton was called to her, thirty-hours after the accident. He passed his index fingers into the mouth, to the anterior edge of the angle of the jaw, and carried them upwards until they reached the coronoid process placed in front of the malar bone. Having ascertained the exact position of parts he seated the patient in a chair, and placing a thumb against each coronoid process, desired her to open her mouth yet wider, for the purpose of relaxing the elevators of the jaw; employing at this moment a slight backward pressure, he at once reduced the dislocation. In the autopsy of a person dying from other causes, after a recent dislocation of the jaw, the accident was easily reproduced, and the exactitude of the anatomical description confirmed.—*Brit. and For. Med. and Chir. Rev.*

